# Gamma-H2AX foci in cells exposed to a mixed beam of X-rays and alpha particles

**DOI:** 10.1186/2041-9414-3-8

**Published:** 2012-11-02

**Authors:** Elina Staaf, Karl Brehwens, Siamak Haghdoost, Joanna Czub, Andrzej Wojcik

**Affiliations:** 1 Centre for Radiation Protection Research, Department of Genetics, Microbiology and Toxicology, Stockholm University, Svante Arrhenius väg 20C, Stockholm, 106 91, Sweden; 2 Institute of Physics, Jan Kochanowski University, Kielce, Poland; 3 Department of Radiobiology and Immunology, Institute of Biology, Jan Kochanowski University, Kielce, Poland

**Keywords:** Ionizing radiation, LET, Alpha particles, X-rays, Mixed beam, Gamma-H2AX, Foci, IRIF

## Abstract

**Background:**

Little is known about the cellular effects of exposure to mixed beams of high and low linear energy transfer radiation. So far, the effects of combined exposures have mainly been assessed with clonogenic survival or cytogenetic methods, and the results are contradictory. The gamma-H2AX assay has up to now not been applied in this context, and it is a promising tool for investigating the early cellular response to mixed beam irradiation.

**Purpose:**

To determine the dose response and repair kinetics of gamma-H2AX ionizing radiation-induced foci in VH10 human fibroblasts exposed to mixed beams of ^241^Am alpha particles and X-rays.

**Results:**

VH10 human fibroblasts were irradiated with each radiation type individually or both in combination at 37°C. Foci were scored for repair kinetics 0.5, 1, 3 and 24 h after irradiation (one dose per irradiation type), and for dose response at the 1 h time point. The dose response effect of mixed beam was additive, and the relative biological effectiveness for alpha particles (as compared to X-rays) was of 0.76 ± 0.52 for the total number of foci, and 2.54 ± 1.11 for large foci. The repair kinetics for total number of foci in cells exposed to mixed beam irradiation was intermediate to that of cells exposed to alpha particles and X-rays. However, for mixed beam-irradiated cells the frequency and area of large foci were initially lower than predicted and increased during the first 3 hours of repair (while the predicted number and area did not).

**Conclusions:**

The repair kinetics of large foci after mixed beam exposure was significantly different from predicted based on the effect of the single dose components. The formation of large foci was delayed and they did not reach their maximum area until 1 h after irradiation. We hypothesize that the presence of low X-ray-induced damage engages the DNA repair machinery leading to a delayed DNA damage response to the more complex DNA damage induced by alpha particles.

## Background

Ionizing radiation is a model agent for studying the mechanisms responsible for genomic stability of cells. Cells can be irradiated with sparsely ionizing X or gamma rays (referred to as low linear energy transfer (LET) radiation) or with densely ionizing alpha particles or heavy ions (referred to as high LET radiation). Low LET radiation mainly induces dispersed damage while high LET radiation gives rise to clustered damage along the particle track. Few studies exist where cells are irradiated with a mixed beam of both radiation types. The action of mixed beams is interesting because it is not known if the two radiation types act in an independent or an interacting manner. Generally speaking, a synergistic (interacting) action of two agents on the cell can primarily occur via two mechanisms: through potentiating the level of damage or through impairing the cellular mechanisms of damage repair. A good example of the former is the interaction of oxygen with ionizing radiation [1-3], while a good example of the latter is the interaction of metals with ionizing radiation [4-7]. In the case of combined action of two radiation types there is no reason to assume that the level of initial DNA damage differs from additivity, because the level of damage is directly proportional to the amount of energy absorbed by the cell. However, it is possible that the simultaneous action of the two radiation types leads to a change of damage quality (an increased damage complexity within chromosome domains) or that the damage induced by one radiation engages the DNA repair machinery to such an extent that the damage induced by the second radiation is not repaired properly.

The action of mixed beams on cells is not only interesting from the perspective of cell biology but also for radiation protection. There is growing concern regarding exposure of cancer patients to mixed beams of low and high LET radiation during radiation therapy. In external beam radiation therapy background neutrons are generated in linear accelerators operating at energies above 10 MeV [8,9], giving rise to neutron equivalent doses per unit photon tumour dose from 0.1 mSv Gy^-1^ to 20.4 mSv Gy^-1^[10]. In fast neutron therapy patients are exposed to photons from the thermalisation of neutrons [11] and in boron neutron capture therapy the therapeutic dose (of about 50 Gy) received by the tumour is composed of a mix of He- plus Li-ions generated during the ^10^B(n,α)^7^Li reaction and of photons generated during the (n,γ) reaction [12,13]. Mixed beam exposures are also not uncommon in our environment. There are urban areas where high indoor radon levels are combined with elevated background gamma radiation to generate absorbed doses of 20 mSv y^-1^ or more, well above the average of 2.4 mSv y^-1^ for the world [14]. Finally, during airplane- and spaceflights high LET cosmic radiation interacts with the shielding material of the cabin to produce gamma background radiation that acts in combination with the high LET particles [15,16].

In the studies on the effect of mixed beam irradiation published so far, both additivity [17-21] and synergism [22-29] have been observed. The main endpoints employed in these studies were clonogenic survival [17,18,21-25,27,28], the micronucleus assay [19,26] and the chromosomal aberration test [20,29]. Interestingly, no studies investigating DNA damage and repair after mixed beam exposure were performed.

The gamma-H2AX assay is well suited for studying the induction and repair of DNA double strand breaks (DSB). The number of gamma-H2AX ionizing radiation induced foci (IRIF) has been observed to be proportional to the number of double-strand breaks produced [30-33]. The existing differences in ionization density and track structure for the actions of radiations of high and low LET [34] are reflected in IRIF characteristics. IRIF after low LET radiation tend to be small and evenly distributed in the cell while IRIF after high LET radiation are larger and clustered in tracks [35-39]. It should be noted that alpha particles induce low as well as high LET damage in cells, due to the presence of delta electrons. In addition to the particle track itself, observed as a string of IRIF if viewed from the side [36,40], the delta electrons induce less complex damage at a distance from the track [41,42], giving rise to smaller IRIF. The possibility to distinguish between IRIF generated by low and high LET radiation on the basis of IRIF size has been discussed previously [36,43,44], but no IRIF results after simultaneous exposures have been published to date.

We have recently developed a dedicated exposure facility that allows studying the cellular effects of mixed beam exposure [45]. The facility allows a simultaneous irradiation of cells with alpha particles from above and X-rays from below and we use it to investigate the formation and disappearance of IRIF in cells exposed to mixed beams. The results indicate a difference in repair kinetics of IRIF induction and repair between the three types of irradiation. However, since the IRIF level after 24 h was similar for all irradiation schemes, long-term effects were not clear. The dose response to mixed beam irradiation was additive.

## Results

### Doses and IRIF classification

For dose response analysis, cells were exposed to 0.13, 0.27 and 0.32 Gy alpha particles, 0.20, 0.40 and 0.80 Gy X rays and 0.27, 0.53 and 0.80 Gy mixed beams, with 25% of the dose from alpha particles. The rational for using these doses was that in our earlier study we saw a synergistic effect of mixed beams in this dose range [46]. For analysis of repair kinetics 0.27 Gy alpha particles, 0.8 Gy X-rays and 0.13 + 0.40 Gy mixed beams (of alpha particles and X-rays, respectively) were the chosen doses. Predicted mixed beam values were obtained by dividing the alpha particle- and X-ray values by half and adding them. The same approach was applied for the lowest dose in the dose response curve (0.27 Gy), while the two highest doses were predicted by adding the full values from the single X-ray and alpha particle exposures. IRIF were analyzed by a module written in software ImageJ, version 1.43 u. Since irradiations as well as image capture were carried out perpendicularly to the glass slide to which cells were attached, one particle traversal was visualized as one large IRIF. IRIF were classified as small or large (SF or LF) based on size. See Methods for further information.

### Program performance and representative images

**Figure 1:**
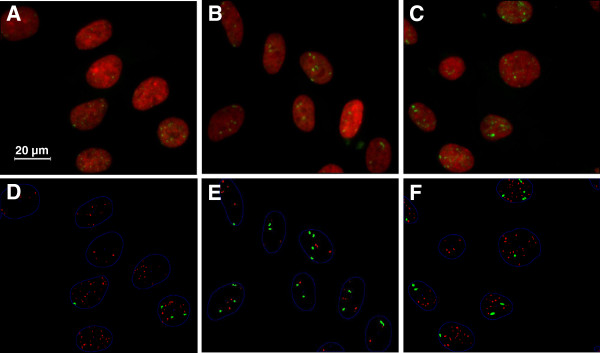
**Original and program output sample images for X-ray, mixed beam and alpha-particle irradiated VH10 cells.** Images were captured 1 h after irradiation. **A**-**C**: Original images. **A**: 0.20 Gy X-rays, **B**: 0.27 Gy alpha particles, **C**: 0.27 Gy mixed beams (0.20 Gy X-rays + 0.07 Gy alpha particles), **D**-**F**, corresponding program output images. **D**: Analysis image from A, X-rays. **E**: Analysis image from **B**, alpha particles. **F**: Analysis image from **C**, mixed beams. In images **D**-**F**: red dots = small foci, green dots = large foci.

In Figure 1 original images as well as output images from the analysis are presented. 1 h after exposure, similar doses of X-rays, alpha particles and mixed beams (0.20, 0.27 and 0.20 + 0.07 Gy respectively) gave rise to different response characteristics. X-rays (Figure 1D) induced mainly SF (red dots in Figure 1D-F) while LF (green dots in Figure 1D-F) made up a larger proportion of the total number of IRIF observed in the nuclei of alpha particle-irradiated cells (Figure 1E). Mixed beam-irradiated cells exhibited an intermediate response (Figure 1D-F).

### IRIF repair kinetics and dose response

**Figure 2:**
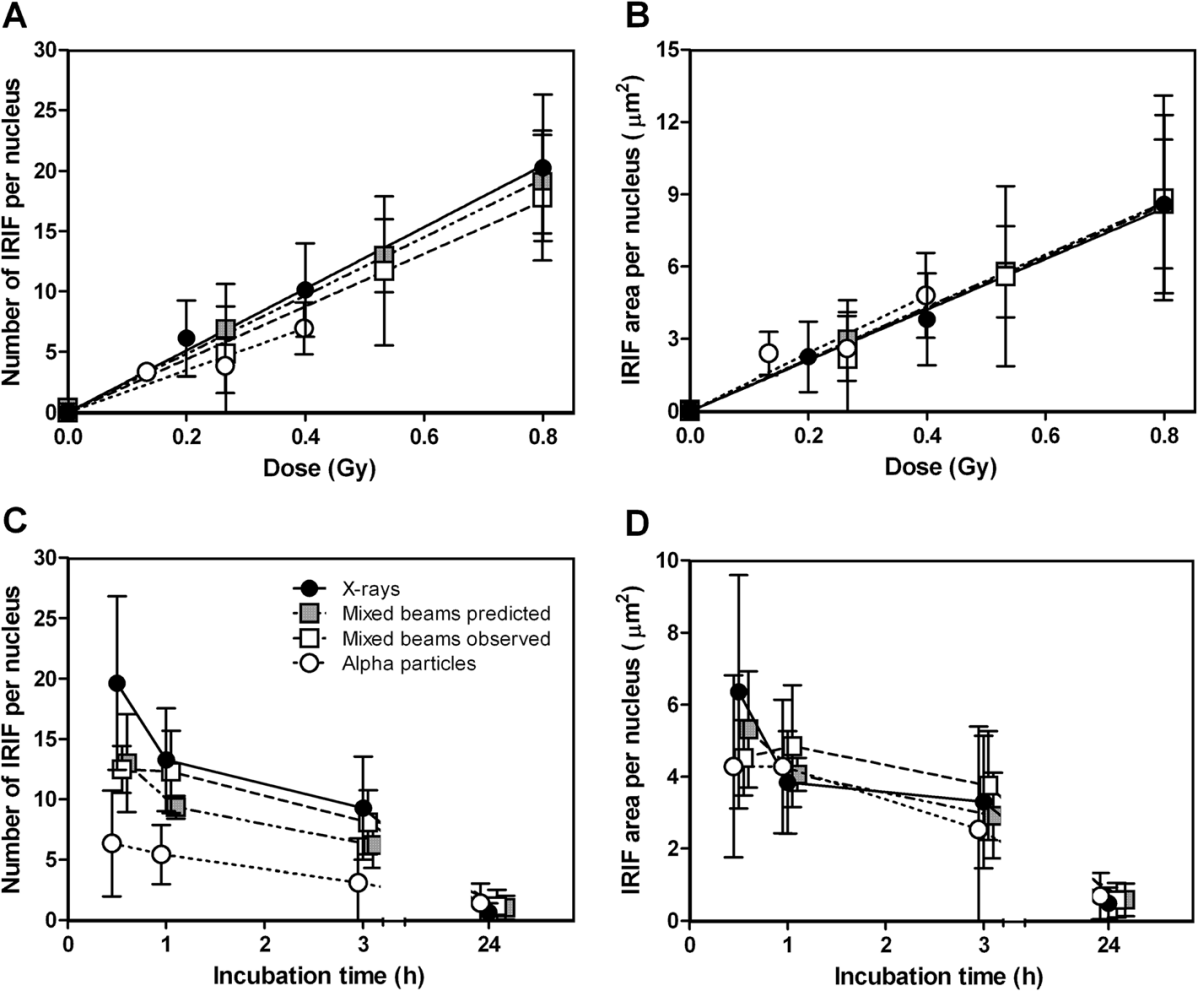
**Dose response and repair kinetics for summarized gamma-H2AX foci number and total area per nucleus.A**: number of IRIF per nucleus for dose response. **B**: IRIF area per nucleus for dose response. **C**: number of IRIF per nucleus for repair kinetics. **D**: IRIF area per nucleus for repair kinetics. Black circles = X-rays, white circles = alpha particles, white squares = mixed beam of 75% X-rays and 25% alpha particles, grey squares = predicted values for mixed beam (assuming additivity). Error bars represent standard deviations. Doses for repair kinetics: X-rays = 0.8 Gy. Alpha particles = 0.27 Gy. Mixed beams = 0.53 Gy. Dose response was scored 1 h after irradiation.

The dose response at 1 h post exposure revealed no significant differences between the exposure schemes. A relative biological effectiveness (RBE) of 0.76 with a standard deviation of 0.52 was observed for alpha particles compared to X-rays (Figure 2A-B). The RBE was calculated based on the difference in slopes for alpha particle relative to X-ray dose response curves for number of IRIF per nucleus. The relatively low RBE originates from the characteristics of the irradiation setup, where one particle track will be viewed as one LF, thereby underestimating the number of IRIF after alpha particle exposure. The dose response for alpha particle-irradiated cells was slightly steeper for IRIF area per nucleus than for number of IRIF per nucleus. The R^2^ for IRIF linear fit was 0.75 for alpha particles, 0.71 and 0.89 for mixed beams (observed and predicted respectively) and 0.82 for X-rays.

For repair kinetics, analysis was carried out 0.5, 1, 3 and 24 h post-exposure. The number and area of IRIF per nucleus in X-ray-irradiated cells decreased steadily with time, significantly so for 0.5 compared to 3 h (p = 0.038), and for 0.5 and 1 h compared to 24 h (p < 0.002 for both). In mixed beam-irradiated cells a significant decrease from 0.5 to 3 h was observed (p = 0.037, Figure 2C). There were no significant changes after alpha particle exposures during the first three time points (Figure 2C-D). The number and area of IRIF per nucleus in mixed beam-irradiated cells were predicted to decrease from 0.5 to 1 h, while the observed response remained unchanged (Figure 2C-D). However, due to overlapping standard deviations this difference was not significant. The total IRIF area per nucleus for alpha particle and mixed beam-irradiated cells was very similar to that of X-rays, indicating that each IRIF, on average, was larger when alpha particles had contributed to the dose (Figure 2D). At the 24 h time point there were no differences between the exposure schemes, neither for number of IRIF, nor the IRIF area per nucleus.

The results for number and area of SF per nucleus were very similar to those depicted in Figure 2 (since the majority of IRIF were small). The same was true for intensity, which was always directly proportional to area. The results for SF and intensity were therefore not shown.

### LF repair kinetics and dose response

**Figure 3:**
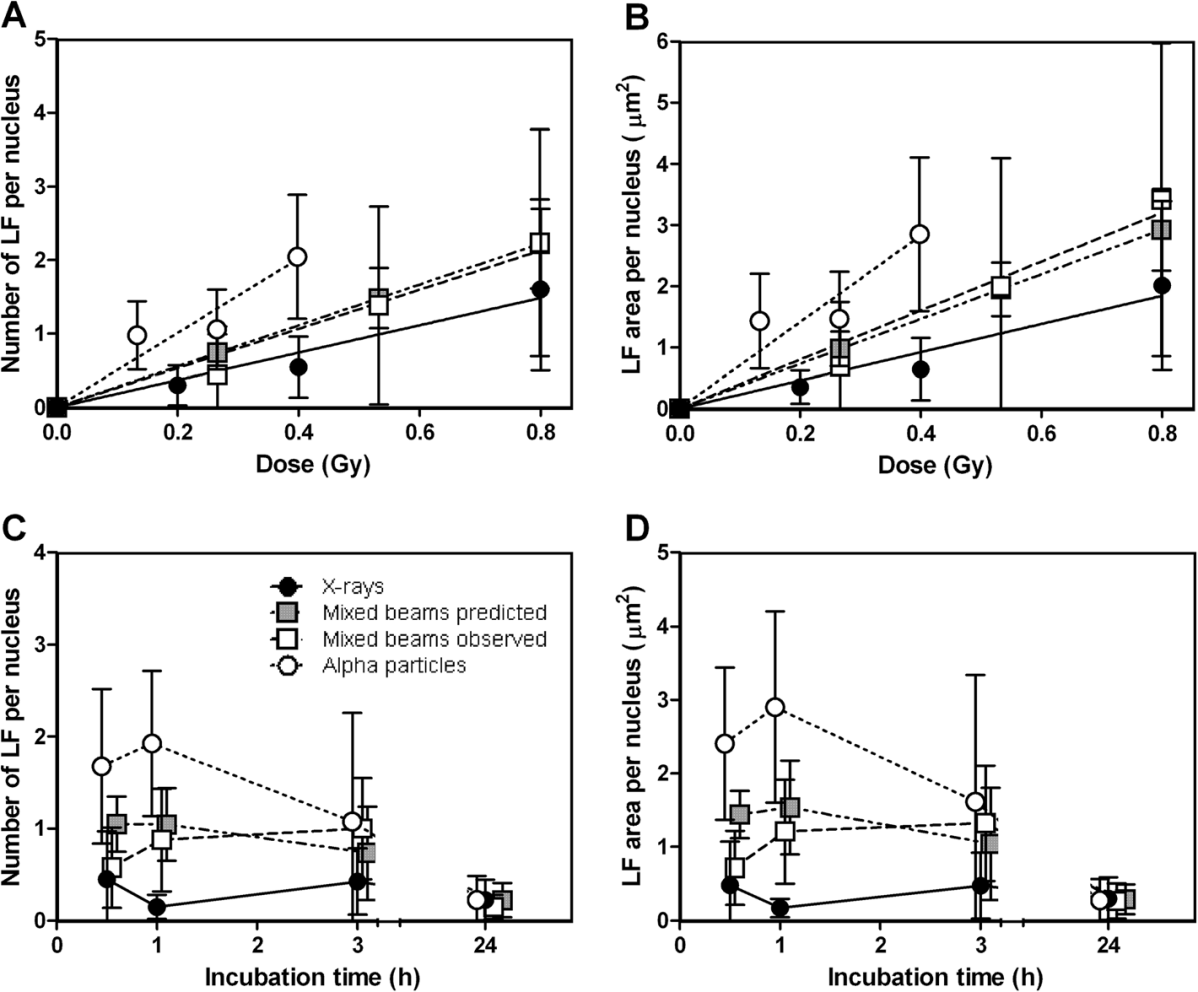
**Dose response and repair kinetics for large gamma-H2AX foci number and total area per nucleus.A**: number of LF per nucleus for dose response. **B**: LF area per nucleus for dose response **C**: number of LF per nucleus for repair kinetics. **D**: LF area per nucleus for repair kinetics. Black circles = X-rays, white circles = alpha particles, white squares = mixed beam of 75% X-rays and 25% alpha particles, grey squares = predicted values for mixed beam (assuming additivity). Error bars represent standard deviations. Doses for repair kinetics: X-rays = 0.8 Gy, Alpha particles = 0.27 Gy. Mixed beams = 0.53 Gy. Dose response was scored 1 h after irradiation.

The dose response for number and area of LF per nucleus revealed a significant difference between the slopes of X-ray and alpha particle dose response curves (p = 0.015 and p = 0.01 for LF number and area respectively, see Figure 3A-B), giving an alpha particle RBE of 2.54 with a standard deviation of 1.11 for LF. Results for mixed beam-irradiated cells were intermediate, with overlapping observed and predicted values, indicating an additive response. The R^2^ for LF linear fit was 0.66 for alpha particles, 0.46 and 0.86 for mixed beams (observed and predicted respectively) and 0.57 for X-rays.

Due to large overlapping standard deviations there were no significant differences in the LF kinetics. At the 24 h time point the numbers of LF were at low, comparable levels for all irradiation schemes. However, two trends could be seen. First, a slight decrease in the number and total LF area in the nuclei of alpha particle-irradiated cells from 1 to 3 h indicated the initiation of dephosphorylation and/or histone removal, a trend that was not observed after mixed beam and X-ray exposure (Figure 3C-D). Second, in mixed beam irradiated cells the number and area of LF were predicted to decrease slightly during the first three time points, while the observed data instead indicated an increase.

Due to the configuration of the setup (alpha particle irradiation from above, and image capture from the same viewpoint), a LF observed in a cell irradiated with alpha particles was expected to depict one particle track. To validate this claim, the fluence was utilized. With an average fluence of 23789 ± 4564 particles per second per cm^2^ and an average DNA area of ~250 μm, for a 60 second exposure (0.27 Gy) the fluence equalled ((23789 ± 4564)/10^8)*250*60 = 3.57 ± 0.68 particles per nucleus. This corresponded well with the number of LF observed in alpha particle-irradiated cells for repair kinetics (Figure 3C), but the LF numbers for dose response was significantly lower (Figure 3A).

### Average SF and LF area

**Figure 4:**
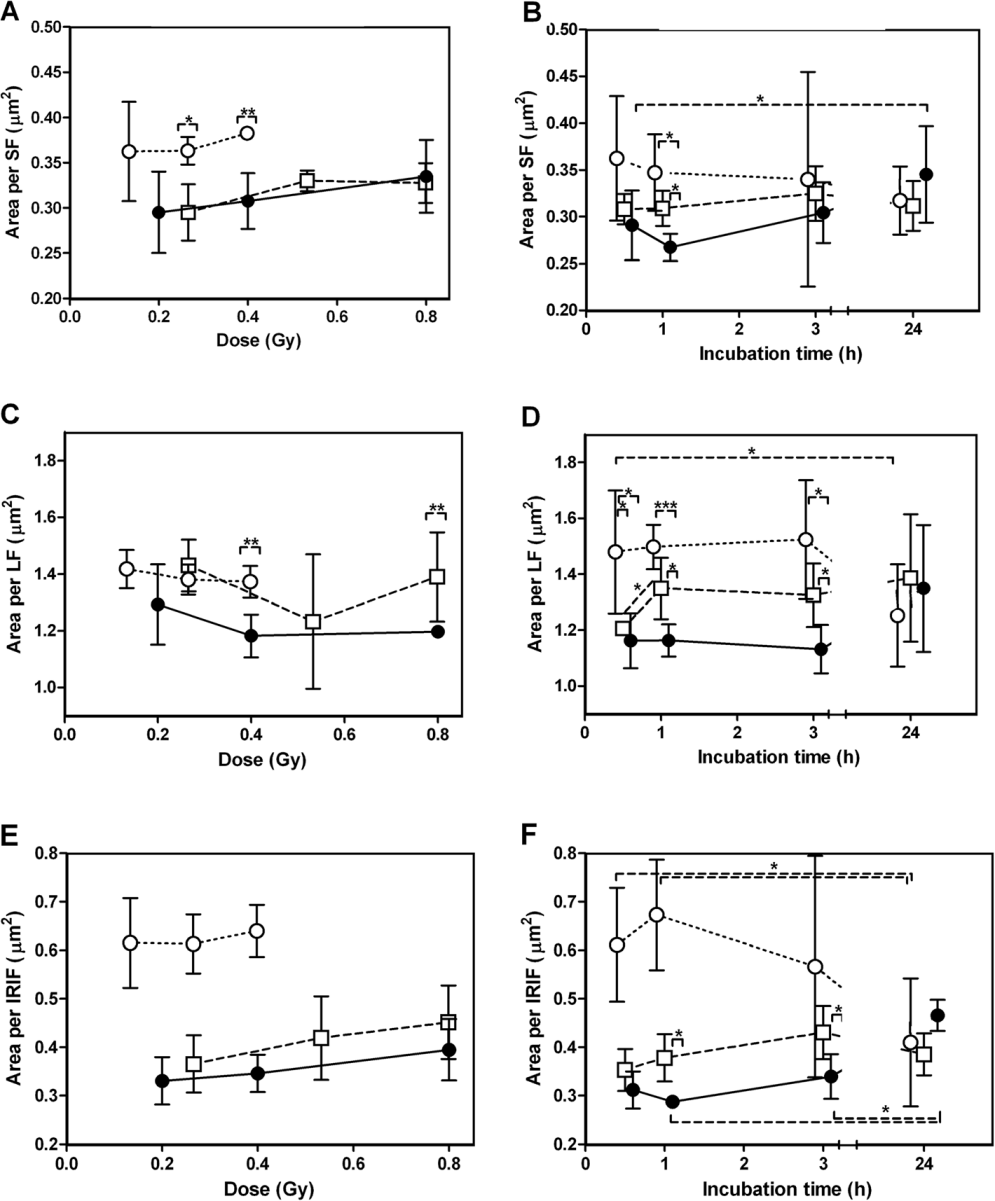
**Average area per individual ionizing radiation-induced gamma-H2AX focus (small, large and summarized).A**: average area per LF for dose response. **B**: average area per SF for repair kinetics. **C**: average area per LF for dose response. **D**: average area per LF for repair kinetics. **E**: average area per IRIF for dose response. **F**: average area per IRIF for repair kinetics. Black circles = X-rays, white circles = alpha particles, white squares = mixed beam of 75% X-rays and 25% alpha particles. Error bars represent standard deviations. Dose response was scored 1 h after irradiation. Doses for repair kinetics: X-rays = 0.8 Gy, Alpha particles = 0.27 Gy. Mixed beams = 0.53 Gy.

The dose response data at 1 h revealed that the average SF in cells irradiated with alpha particles was larger than their X-ray- and mixed beam-induced counterparts (significantly so at the two highest doses, p = 0.018 compared to mixed beams at 0.27 Gy and p = 0.003 compared to X-rays at 0.40 Gy, Figure 4A). In the repair kinetics study an individual SF was generally largest in alpha particle-irradiated cells, intermediate in mixed beam- and smallest in X-ray-irradiated cells for the first three time points (Figure 4B). X-ray-induced SF were significantly smaller than alpha particle- and mixed beam-induced SF 1 h after irradiation (p = 0.011 and p = 0.013 respectively), and the average area only changed with time for X-ray-irradiated cells (significantly larger at 24 h compared to 0.5 h, p = 0.027).

The LF area was not dose-dependent 1 h after exposure, at least not for the investigated doses where IRIF overlap was avoided (see Figure 4C). Significant differences were observed for alpha particle- and X-ray-irradiated cells at 0.40 Gy and mixed beam- and X-ray-irradiated cells at 0.80 Gy (p = 0.007 and p = 0.002 respectively). The average area per LF for repair kinetics was significantly larger in alpha particle-irradiated cells than in X-ray-irradiated cells (Figure 4D, p = 0.040, p < 0.001 and p = 0.014 for 0.5, 1 and 3 h respectively). Mixed beam-irradiated cells generally had smaller LF that alpha particle-irradiated cells (significant for 0.5 h, p = 0.048) and larger LF than X-ray-irradiated cells (significant at 1 and 3 h, p = 0.024 and 0.035 respectively). The area of an average LF in mixed beam-irradiated cells increased significantly from 0.5 to 1 h (p = 0.042).

When adding the results and regarding both SF and LF as simply IRIF, the average area per IRIF was significantly larger for alpha particle-irradiated cells than the other irradiations at 0.5 and 1 h (p < 0.001, Figure 4E-F). For dose response, a non-significant dose-dependent increase in the average area per IRIF for mixed beam- and X-ray-irradiated cells was observed (Figure 4E). For repair kinetics, no significant time-dependent trends were observed for mixed beam-induced IRIF, but they were significantly larger than X-ray-induced IRIF at 1 and 3 h after exposure (p = 0.01 and 0.045 respectively, Figure 4F). X-ray-induced IRIF were significantly larger at 24 h than at earlier time points (p < 0.001 for 0.5 and 1 h, p = 0.004 for 3 h), while the opposite was true for IRIF after alpha particle-irradiation (p = 0.02 for 1 compared to 24 h, Figure 4F).

### Relative LF frequency and area in mixed beam-exposed cells – investigating the repair kinetics difference

**Figure 5:**
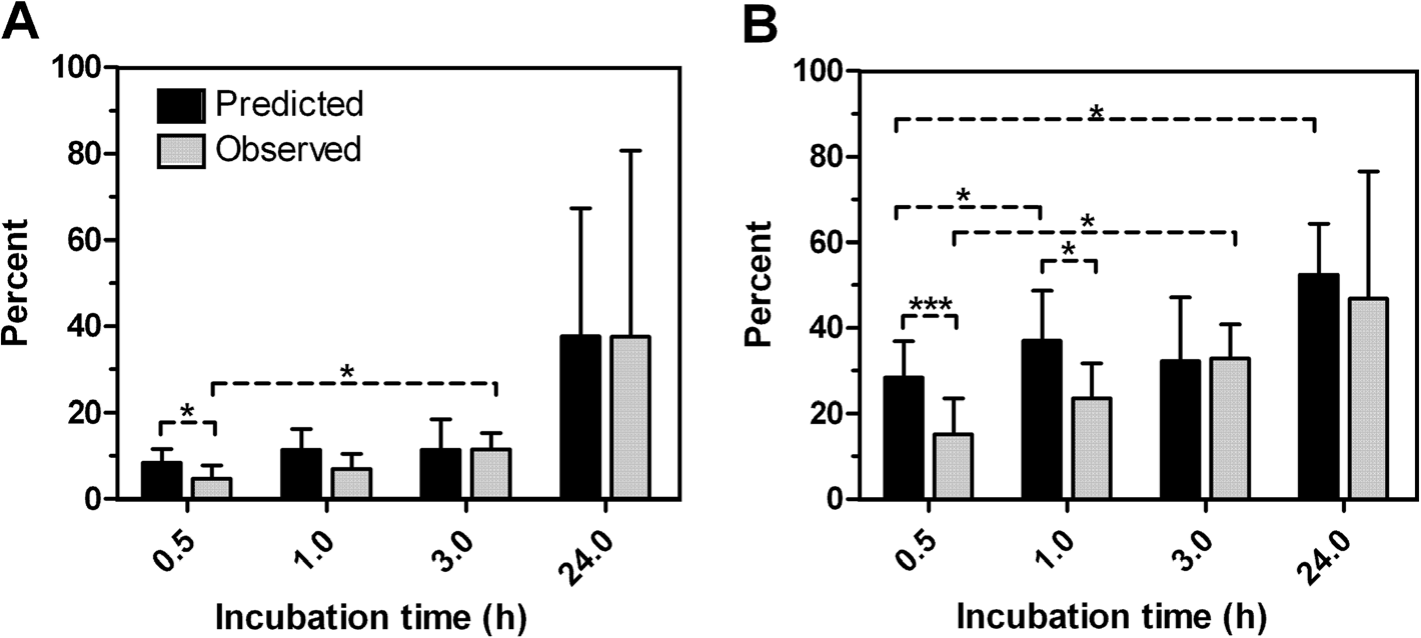
**Observed and predicted relative frequency and area of large gamma H2AX foci per nucleus.A**: Relative LF number. **B**: Relative LF area. Black bars = Predicted. Gray bars = Observed. * = significant, p ≤ 0.05. *** = significant, p ≤ 0.001. Error bars represent standard deviations. Mixed beams dose = 0.53 Gy (0.4 X-rays + 0.13 alpha particles).

Large IRIF were investigated further by calculating the LF contribution in percent to the total IRIF (hereafter called relative LF contribution). For dose response there was only one significant difference between observed and predicted; at 0.27 Gy the predicted LF frequency of total number of IRIF was significantly larger than the observed (p = 0.034). Since this difference was not observed for the area there were no consistent trends for the dose response data, and the results did not merit being shown as a figure. For repair kinetics however the observed relative LF frequency was significantly higher at 3 h as compared to 0.5 h after irradiation (Figure 5A). The same trend was present at 1 h, but was not significant. Analogously to the relative LF frequency, the observed relative LF area was significantly lower than predicted 0.5 h after exposure (p < 0.001), and at the 1 h time point the trend observed in Figure 5A was significant (p = 0.039, Figure 5B). In addition, the observed relative LF frequency and area were significantly higher at 3 h after irradiation as compared to 0.5 h (p = 0.033 and p = 0.021 respectively), indicating a steady increase in the LF proportion of total IRIF number and area with time (Figure 5A-B). Predicted relative LF area was significantly larger at 1 h and 24 h than 0.5 h after irradiation (p = 0.032 and 0.023, respectively).

## Discussion

The aim of the study was to evaluate the effect of a mixed beam of alpha particles and X-rays on the level of IRIF in VH10 cells. When the dose response 1 h after exposure was studied, no interaction between the two radiation types was observed. However, the repair kinetics for mixed beam-irradiated cells differed between observed and predicted.

As observed in Figure 1, IRIF could be grouped in two categories: large foci (LF) and small foci (SF). Previously, Costes et al. [43] and Leatherbarrow et al. [36] observed a difference in IRIF size in cells exposed to low and high LET radiation. Bracalente et al. [44] also noted LF and SF in cells irradiated with gamma radiation and Li ions and concluded that IRIF size is a marker allowing discriminating the effects of radiations of different LET. As mentioned in the introduction, alpha particles induce high as well as low LET damage. In addition to the particle track [36,40] delta electrons induce IRIF outside of the track [41,42]. This phenomenon, along with the beta component of the ^241^Am source (dose rate 0.025 Gy/min) accounted for the mixture of LF and SF observed in alpha particle-irradiated cells. Due to the orthogonal configuration of the irradiation setup, each LF in alpha particle-irradiated cells was expected to represent one particle traversal. As noted in the results section, the theoretical number of traversals in cells irradiated with alpha particles (as calculated based on fluence) corresponded well with the number of LF observed in alpha particle-irradiated cells (Figure 4B), and confirmed the theory that the majority of LF in alpha particle-exposed cells represented alpha particle tracks. The lower number of LF observed in the dose response experiments as compared to repair kinetics experiment may be due to the fact that the former cells were slightly smaller than the latter. The reason for the difference is, however, not clear.

Since no collimator was used, paths of particles traversing the cell nucleus at steep angles, resembling “streaks” of IRIF, were expected to occur, as reported by Leatherbarrow et al. [36]. Interestingly, such IRIF were never observed. A similar result was reported by Costes et al. [43]. This suggests that steep-angle alpha particles from our orthogonal irradiation system do not reach the cell layer (due to the increased travelling distance through air and Mylar) or alternatively, reach the cells but, once there, do not have enough energy to induce more than one IRIF.

It is known that the repair of DSB is slower when induced by high, relative to low LET radiation [36,47] and is thought to reflect the presence of clustered DNA damage induced by high LET radiation [48,49]. This difference was seen in our results as a faster disappearance rate of IRIF in the nuclei of X-ray-exposed cells as compared to cells exposed to alpha particles. For dose response the curves overlapped, giving a RBE of 0.76 ± 0.52 for alpha particles compared to X-rays. The same result, a RBE = 1.0 ± 0.3, has previously been observed by Franken et al. [50] who used a setup similar to ours, where cells were irradiated orthogonally. Recently, Du et al. performed a 3D analysis of carbon ion IRIF tracks, and observed about 15 IRIF per μm [51]. In the setups applied by us and Franken et al. [50] one particle track could only be viewed as a singe IRIF, which led to an underestimation of the number of IRIF after alpha particles and thereby the RBE. Previously, alpha particle RBE values of 2.57 and 1.90 for 37% and 10% clonogenic survival in AA8 cells and 3.2 for micronuclei in human peripheral blood lymphocytes have been observed using this setup [45,46]. The dose response of mixed beam-irradiated cells was intermediate to that of X-ray and alpha particle-irradiated cells, and the differences in repair kinetics between observed and predicted for the first 3 time points were non-significant (Figure 2C-D). 24 h after exposure all cells, regardless of exposure scenario, contained similar (low) levels of IRIF. This somewhat unexpected result is most likely due to the relatively low doses employed in this investigation.

However, when LF were investigated, the differences between alpha particles and X-rays became more pronounced, with a larger LF presence and a RBE of 2.54 ± 1.11 for alpha particles (Figure 3C-D). Kinetics indicated an earlier initiation of dephosphorylation of LF in the nuclei of alpha particle-exposed cells compared to in cells exposed to mixed beams. When comparing observed and predicted mixed beam exposures two clear trends were observed: the frequency and area of LF were initially lower than predicted and increased during the first 3 hours of repair (while the predicted values did not, Figure 3C-D). By plotting the LF contribution as percent of the number and area of IRIF, these trends were confirmed to be significant (Figure 5A-B). Moreover, the average area of an individual LF significantly increased from 0.5 to 1 h, an effect neither observed in X-ray- nor alpha particle-irradiated cells, nor for SF (Figure 4D, 4B for SF). The analysis of IRIF and LF distributions revealed that this trend was not due to the presence of a few outliers, but was representative for the whole cell population (data not shown). Taken together these results indicate that the formation of H2AX foci at sites of complex DNA damage represented by LF was slower in mixed beam-exposed cell nuclei, as compared to exposure to X-rays and alpha particles alone. The observation that LF in mixed beam-exposed cells tended to disappear at a slower rate than in alpha particle-exposed cells can be interpreted as a manifestation of an interaction between low and high LET radiation. It should be stressed that other interpretations of this observation are possible, for example based on the recent finding suggesting merging of foci to form repair factories [42] or the non-linear kinetics for H2AX phosphorylation and dephosphorylation [52]. It can be speculated that an interaction between the low and high LET component is due to two mechanisms: a changed damage quality (increased damage complexity) or an impaired DNA repair. The first possibility appears unlikely, as clustered DNA damage is produced almost exclusively within single radiation tracks so that the probability of overlap of separate tracks over these small dimensions is negligible even at very high doses [48]. Moreover, an increased damage complexity was not observed in cells exposed to mixed beams and analysed 1 h post radiation (dose response experiments). It thus appeared more likely that exposing cells to a mixed beam of high and low LET radiation resulted in an impairment of DSB repair. However, although the DNA damage response after mixed beam exposure was slower than expected during the first 3 h, 24 h after exposure the levels of IRIF and LF were similar for all exposure schemes. In this investigation, the conclusion is therefore that mixed beams results in a delayed DNA damage response, rather than an impaired DNA repair.

IRIF are formed by phosphorylation of the H2AX histone and disappear as a consequence of dephosphorylation [43]. It appears possible that the presence of low LET radiation-induced damage, distributed evenly in the cell nucleus, engages the responsible kinases [53] to such an extent, that the phosphorylation of histones at sites of complex, high LET radiation-induced damage is delayed. The consequences of this delay are not clear, since 24 h after irradiation the levels of IRIF were similar for all irradiation schemes. However, it is worthwhile to note that our earlier study showed a higher than predicted level of micronuclei in mixed beam-exposed human peripheral blood lymphocytes [46]. There we also speculated that DNA repair is impaired in cells exposed to a mixed beam. This mechanism could furthermore account for the synergistic effect of high and low LET radiation observed by others [22-29]. Why the synergistic effect is not always seen [17-21], and under which conditions it may appear is presently not known and requires further studies.

Apart from assuming that there is an interaction between alpha particles and X-rays, the observation that LF in mixed beam exposed cells tended to disappear at a slower rate than in alpha particle-exposed cells can also be accounted for by the non-linear kinetics for H2AX phosphorylation and dephosphorylation [52] as well as imprecision in the image capture and analysis steps. The latter explanation appears to be less probable in view of the good reproducibility of the observation.

The kinetics of IRIF formation and disappearance observed by us are generally in good agreement with the results published by others. The kinetics of IRIF disappearance in normal human fibroblasts exposed to low LET irradiation has previously been observed to follow a two-phase pattern, with a fast and a slow component [54-59]. In this investigation the decrease was steeper from 0.5 to 1 h than from 1 h to 3 h, indicating that a biphasic response is probable. The value at the first time point (30 min after irradiation) was 19.6 ± 7.2 IRIF per nucleus for 0.8 Gy and thus 24.5 ± 9.0 IRIF per nucleus per Gy (for the dose response at 1 h this value was observed to be 25.3 ± 4.5 IRIF per nucleus per Gy). This is in good agreement with previously published gamma-H2AX data in human fibroblasts, where 19 [36] as well as 24 [56] and up to 50 [59] IRIF per nucleus per Gy have been observed 30 min after low LET irradiation. 15 min after low LET irradiation 25 [55] and 37 [54] IRIF per nucleus per Gy were observed. The relatively modest decrease in IRIF per nucleus from 30 min to 1 h for X-ray irradiated cells (Figure 2C) was smaller than previously published differences between maximal number of IRIF and the 1 h time point [36,54,56], indicating that the IRIF peak may have appeared before the 30 min time point. Other factors such as the staining procedure or the image capturing and analysis procedure may also have contributed to the observed differences. The kinetics of IRIF disappearance in cells exposed to alpha particles (Figure 2C), was somewhat slower than that recorded by Riballo et al. [55] and Leatherbarrow et al. [36], but similar to the results of Schmid et al. [60] and Costes et al. [43]. It should also be noted that the cells were neither confluent nor otherwise synchronized. The large standard deviations (especially for LF) and the relatively high number of foci in un-irradiated cells (1.45, standard deviation 0.91) could have been influenced by the fact that the cells were not in the same cell cycle phase. However, the cycling cell populations in our bodies (for example the peripheral blood lymphocytes) are generally among the most sensitive to ionizing radiation, and conducting experiments with cycling cells is therefore a valid approach.

It is interesting that the average area of an IRIF (and SF) tended to increase with dose for X-ray and mixed beam-irradiated cells, but not for cells exposed to alpha particles (Figure 4E). This could be due to overlapping of small IRIF or increasing complexity of DSB with increasing dose [48]. If overlapping IRIF was the cause, a saturation of the dose response curve for IRIF number would occur – an effect that indeed was observed following doses X-ray exceeding 1 Gy (data not shown). An interesting hypothesis that also explains the saturation of the dose–response curve at high doses was recently suggested by Neumaier et al. [42]. These authors observed that, following exposure of human mammary epithelial cells to high doses of radiation, IRIF clustered into repair centers (also called repair factories). Which explanation applies to our cells is not known and a closer examination would require the use of confocal 3D microscopy [42,61], which is however beyond the scope of the present investigation.

It should be noted that due to a higher dose rate of the alpha source as compared to the X-ray source the exposure to mixed beam was not absolutely synchronized: irradiation was started simultaneously, but exposure to alpha particles was terminated ahead of exposure to X-rays. This exposure scenario can be considered as partly sequential. Interestingly, it has recently been observed that, in cells exposed to a sequential beam of low LET protons and high LET heavy ions, IRIF behave differently depending on the order in which the exposures were performed: protons applied before ions acted in a predicted manner, while heavy ions before protons resulted in cells displaying nearly exclusively LF ^a^. The authors explain this result by assuming that the DNA repair capacity of cells exposed to heavy ions is insufficient to cope with damage induced by protons. That cells display both LF and SF after mixed beam irradiation indicates that the recognition of low LET damage is not impaired by the fact that the high LET exposure is terminated earlier.

## Conclusions

In conclusion, the repair kinetics of LF in cells irradiated with a mixed beam of alpha particles and X-rays was significantly different from predicted based on the effect of the single dose components. The initial phosphorylation of the damages along the particle track was slower than expected since LF did not reach their maximum area until 1 h after exposure. This indicated that the presence of low LET damage delayed the DNA damage response to high LET damage. This effect was not detected for the dose response 1 h after irradiation (where a RBE of 0.76 ± 0.52 for IRIF and 2.54 ± 1.11 for LF in alpha particle-irradiated cells was observed) and did not influence the level of residual IRIF or LF 24 h after irradiation.

## Methods

### Cell culturing and irradiation

Primary human VH10 fibroblasts (telomerase-immortalized cells) were obtained from the foreskin of a 10-year old boy [62]. The cells were cultured in DMEM (Dulbecco’s Modified Eagle’s Medium, Sigma-Aldrich, Stockholm, Sweden) supplemented with DBS (Bovine Calf Serum, HyClone, Thermo Fischer Scientific, Göteborg, Sweden) and PEST (Penicillin-streptomycin solution stabilized, Sigma-Aldrich). The cells were re-seeded once per week and kept non-confluent at all times. Cells in passage 8 to 13 were seeded out on 22 x 22 mm sterilized glass cover slips (VWR International AB, Stockholm, Sweden) that were positioned, one per well, in 6-well plates (Thermo Fischer Scientific). The cell density at the day of exposure was 50 000 cells/well, below confluence (see Figure 1 for a visualization of cell density).

Cells on cover slips were irradiated and sham-exposed on round polyamide (PA) discs, 155 mm in diameter. The discs were custom-constructed in the Institute for Energy-JRC, Petten, Netherlands according to a design based on Edwards et al. [63], and are described in detail in Staaf et al. [45]. Before exposure the discs were cleaned with 70% ethanol and kept for 1 h in a 37°C incubator. Each cover slip was positioned cell-side up in the centre of the polyamide disc and covered with ~100 μl DMEM (to avoid drying of cells) and a 1.5 μm Mylar foil lid. On average it took 2 min to remove a cover slip with irradiated cells from the irradiation incubator and then the PA disc, position a fresh cover slip and put the PA disc back into the exposure facility.

### Mixed beam exposure facility

The mixed beam exposure facility MAX consists of an incubator, with the alpha irradiator AIF 08 positioned inside and an X-ray tube positioned underneath [45]. The alpha particle exposure facility was constructed in the Institute of Nuclear Chemistry and Technology, Warszawa, Poland and incorporates a ^241^Am source (AP1 s/n 101, Eckert and Ziegler, Berlin, Germany) and a movable shelf for positioning cells on the PA discs at discrete distances from the source. The source has a total activity of 50 ± 7.5 MBq, an active area of 180 x 180 mm and rotates 2 revolutions per second during exposure.

The fluence and LET of alpha particles was measured with the help of a PADC (polyallyl diglycol carbonate) track-etched detector [64] and was 23789 ± 4564 particles per second per cm^2^[45].

The LET of alpha particles when entering the cell layer ranged from 100 keV/μm to 172 keV/μm. In addition, the LET value also varied from 100 keV/μm to 238 keV/μm depending on the incident particle energy and the thickness of DMEM on top of the cell layer. The SRIM software (http://www.srim.org/) and the approach described by Thomas et al. [65] were used to calculate the dose rate at the entry of alpha particles into the medium, 0.24 Gy/min [45]. No collimator was present during the exposures.

There are two additional dose components of the ^241^Am source: beta and gamma radiation. The maximum energy of the beta radiation did not exceed 70 KeV. Ranges and energy deposition of electrons was determined using the ESTAR software (http://physics.nist.gov/PhysRefData/Star/Text/ESTAR.html), based on the activity of the source. Total dose rate of electrons was calculated to be 25 mGy/min [45]. This dose rate was added to the alpha dose rate and to the dose rate of X-rays in the upper table position (see below). The total dose rate from the alpha source was thus 0.265 Gy/min. The gamma component was measured using a RNI 10/R INTENSIMETER (Unirad AB, Årsta, Sweden) and was found to be below 1 mGy/h at 1 mm from the source. This dose component was therefore omitted in the study.

The X-ray tube (YXLON SMART 200, Yxlon International, Hamburg, Germany) was operated at 190 kV, 4.0 mA without filters. However, before reaching the cells the X-rays were filtered by the copper alloy stainless steel bottom of the incubator and the bottom plate and movable shelf of AIF-08 (12.0 and 8.0 mm aluminium respectively). The dose rate for X-rays was 0.068 Gy/min in the shelf bottom position (X-rays alone) and 0.052 Gy/min in the shelf top position (simultaneous exposure). More details can be found in Staaf et al. [45].

For analyzing repair kinetics, cells were exposed to 0.27 Gy alpha particles, 0.8 Gy X-rays and 0.13 + 0.40 Gy mixed beams (of alpha particles and X-rays). After exposure, cells on cover slips were returned to the medium-filled wells and incubated 0.5, 1, 3 and 24 h at 37°C in a standard incubator with 5% CO_2_. For analyzing the dose response, cells were exposed to 0.13, 0.27 and 0.32 Gy alpha particles (irradiation times: 30 sec, 1 min and 1 min 30 sec), 0.20, 0.40 and 0.80 Gy X rays (irradiation times: 2 min 56 sec, 5 min 51 sec and 11 min 42 sec) and 0.27, 0.53 and 0.80 Gy mixed beam (irradiation times: 15 sec + 2 min 44 sec, 30 sec + 5 min 29 sec and 45 sec + 8 min 13 sec of alpha particles and X-rays respectively) and harvested after 1 h incubation. The 1 h time point was chosen for dose response since both X-ray and alpha particle-induced IRIF were present to a high level at this time. In mixed beam exposures, alpha particles contributed with 25% and X-rays with 75% of the dose. Four independent experiments were performed for analyzing repair kinetics and dose response.

### The gamma-H2AX assay

After exposure and the subsequent incubation cells were transferred to room temperature, fixed in 3% paraformaldehyde, 2% sucrose in PBS (all from Sigma-Aldrich) for 10 min and thereafter permeabilized in 2% Triton X-100 (Merck Chemicals, Darmstadt, Germany) in PBS for 5 min. Anti-phospho-histone H2AX (serine 139) mouse monoclonal primary antibody (Millipore, Billerica, USA), diluted 1:800 (1.25 μg/ml) in PBS supplemented with 2% Bovine Serum Albumin Fraction V (Sigma-Aldrich) was added to cells. Samples were incubated for 30 min in a humid chamber at 37°C. Secondary antibody (anti-mouse IgG conjugated to fluorescent isothiocyanate, FITC, Sigma-Aldrich), diluted 1:200 (5 μg/ml) in PBS supplemented with 2% Bovine Serum Albumin Fraction V (Sigma-Aldrich) was added and samples incubated as before. Counterstaining of nuclei was performed with DAPI (0.000025% in PBS, Sigma-Aldrich) and the samples were incubated 10 min at room temperature in a humid chamber. Cells on cover slips were briefly dried before being mounted on slides with Vectashield (Vector laboratories, Burlingame, Canada) and sealed with rubber glue.

### Image acquisition, analysis of IRIF and classification of SF and LF

Images capture was performed with a setup consisting of a 100x oil immersion objective, a Cool Cube 1 CCD camera (Metasystems, Althusheim, Germany) and a Nikon Eclipse E800 (Nikon, Tokyo, Japan) fluorescence microscope. The ISIS image analysis system (version 5.3, Metasystems) was used for 2D image capture. To avoid saturation of the FITC signal, the image integration time for each experiment was determined based on the automatic integration time measured in cells exposed to the highest dose of radiation and/or showing many IRIF with high intensity. Once determined, the same fixed integration time was applied to the FITC channel for all subsequent images. ISIS automatically determined the optimal integration time for the DAPI channel. The images were exported in TIF format, decompressed with Irfanview (ver. 4.20, Irfan Skiljan, Vienna, Austria) and analyzed with ImageJ (version 1.43u, http://rsbweb.nih.gov/ij/index.html), using a module written by Dr. Niklas Schultz at GMT Department, Stockholm University, Sweden [66]. The program evaluates the DAPI and FITC channels independently, is capable of automatically identifying cell nuclei and register IRIF parameters within the nuclear area. IRIF area, intensity and the number of IRIF per nucleus were given in the output file. When cells are irradiated perpendicularly to the cover slip (as in the present investigation), it is not possible to distinguish individual IRIF within an alpha particle track. Each particle track in alpha particle-irradiated cells would then be viewed as a single IRIF, which would be larger than those observed after low LET irradiation [67]. The program was modified to classify IRIF as large foci (LF) or small foci (SF). After running the program the resulting images were manually inspected and cells with the following characteristics removed: 1) very large (>500 μm^2^) and/or two or more cells overlapping, 2) very small (<90 μm^2^) and/or cut off by the image edge, 3) in metaphase or S-phase. The S-phase cells could be identified by their larger size and the presence of many, but smaller and weaker foci than the IRIF observed in non-S-phase cells. The DNA area of cells accepted for analysis ranged from 90 to 500 μm^2^, with an average DNA area of 249 ± 34 μm^2^ when studying repair kinetics and 238 ± 17 μm^2^ for dose response.

For analysing repair kinetics, 50 cells per dose point were analysed per experiment, except for experiment 1, 1 h X-rays and 3 h mixed beam where 49 and 48 cells were scored, respectively. For dose response, 50 cells per dose point were analysed in the first two experiments and 200 cells per dose point for the subsequent two experiments.

### Statistical analysis

Basic calculations and summaries were performed in Microsoft Office Excel 2003, and plots were constructed in the software Prism (version 5.0, GraphPad, La Jolla, USA). The dose response curves for alpha particles, X-rays and mixed beam (observed and predicted) were fitted with least squares and compared utilizing the function “test whether slopes and intercepts are significantly different” which is based on the analysis of covariance (ANCOVA) and is included in Prism. Since there were no differences between linear and linear-quadratic fits to the dose response data, the less complex linear equation was chosen. For repair kinetics, paired t-tests were used for observed versus predicted results and unpaired t-test was used for comparisons within the raw data

IRIF were divided into LF (large foci) and SF (small foci). Based on visual inspection of sample images, followed by distribution tests for overlapping populations to ensure that the cutoff was correct, an IRIF was classified as SF if the area was 8 – 75 pixels and LF if the area was ≥76 pixels. By using a calibrated measurement scale it was observed that a length of 93 pixels equals 10 μm, and thus 1 pixel = 0.012 μm^2^. For dose response, the predicted values of IRIF parameters in mixed beam-exposed cells (assuming additivity) were calculated by adding dose values obtained from the individually fitted curves for each alpha particle and X-ray experiment. For repair kinetics, predicted values were obtained by dividing by half the values for alpha particles and X-rays (for a corresponding time point) and adding them. The rationale for this approach was that the dose responses were linear, and that single beam component doses used for mixed beam irradiation were equivalent to half the doses used in single-radiation exposures.

One experimental point, 0.27 Gy alpha particles for alpha dose response experiment 2, exhibited significantly higher IRIF numbers and total IRIF intensities per nucleus than the corresponding experimental points for the other three experiments. The Nalimov test for outlier detection was performed, and since the experimental point was classified as an outlier (significance level p = 0.001) it was removed from all calculations and plots.

The number of foci in control cells generally varied between 0.2 and 2.3 foci per nucleus (calculated as the sum of foci in the whole cell population cells divided by the number of cells). In all cases the number of control foci were subtracted from the number of IRIF observed in irradiated samples. One control sample, mixed beam dose response experiment 2, contained 5.3 foci per nucleus, significantly more than the average 1.45 ± 0.91. Why this observation deviated is not clear. Nevertheless, this value was also subtracted.

### Endnotes

^a^Chaudhary, P., Abele, W. H., Moscariello, M., Bennett, P.V. and Sutherland, B.M. Role of **53BP1 Foci Formation in Proton HZE Sequential Dual Ion Beam induced Neoplastic Transformation in Primary Human Fibroblasts**. *Heavy Ions in Therapy and Space Symposium* 2009, July 6–10, Session 9 Cancer; Page-229. 2009, IHK Cologne, Germany. Manuscript submitted to Radiation Research.

## Abbreviations

IRIF: Ionizing radiation-induced foci; LET: Linear energy transfer; LF: Large ionizing-radiation induced foci; PA: Polyamide; RBE: Relative biological effectiveness; SF: Small ionizing radiation-induced foci.

## Competing interests

The authors declare that they have no competing interests.

## Authors’ contributions

AW and ES designed the experiments, ES performed the irradiation, image capture and data analysis, JC performed the dosimetry and wrote the dosimetry section of the materials, KB performed cell culturing and prepared cells for irradiation, ES wrote the manuscript, KB, SH and AW provided critical editing of the manuscript. All authors approve of the final version of the manuscript.
